# Challenges in evaluating cognitive impairment in diabetics in the Democratic Republic of the Congo

**DOI:** 10.1590/1980-5764-DN-2023-0082

**Published:** 2024-09-23

**Authors:** David Shamputi, Célestin Kaputu-Kalala-Malu, Bives Mutume Nzanzu Vivalya, Stella-Maria Paddick, Raj Kalaria

**Affiliations:** 1 Université Evangélique En Afrique, University Teaching Hospital of Panzi, Department of Internal Medicine, Bukavu Town, Republic Democratic of Congo.; 2 University of Kinshasa, Kinshasa University Teaching Hospital, Centre Neuropsychopathologique, Department of Neurology, Republic Democratic of Congo.; 3 Kampala International University-Western Campus, Department of Psychiatry, Bushenyi, Uganda.; 4 Newcastle University, Translational and Clinical Research Institute, Campus for Ageing & Vitality, Newcastle upon Tyne, UK.; 5 University of Nairobi, Faculty of Health Sciences, Nairobi, Kenya.

**Keywords:** Cognitive Dysfunction, Dementia, Diabetes Mellitus, Neuropsychology, Africa, Disfunção Cognitiva, Demência, Diabetes Mellitus, Neuropsicologia, África

## Abstract

Dementia is a global public health issue, with 57.5 million people living with at least one type of dementia in 2019 worldwide, and projected to rise to 152 million by 2050.

**Objective:**

We assessed the cognitive function in diabetic patients aged 60 or older in Bukavu city, in the eastern Republic of the Congo (DRC).

**Methods:**

This case-control study involved 123 patients with established diabetes mellitus (DM) and 123 controls over 60-year-olds also with high rates of illiteracy. Cognitive function was assessed using the Swahili version of the Community Screening Instrument for Dementia (CSI-D).

**Results:**

Foremost, our study revealed language-related differences between Swahili spoken in other eastern African countries such as Tanzania and Kenya, where the Swahili CSI-D is readily applied, compared to the Swahili spoken in Bukavu (DRC). Our results also showed that cognitive impairment was present in 18.7% of the total 246 participants. Remarkably, the prevalence rate of cognitive impairment was higher in the non-diabetic group (12.2 versus 25.2%; p=0.009). Participants aged 80 or older were more likely to present with cognitive impairment compared to those aged less than 80 (adjusted odds ratio — aOR=70.27; 95% confidence interval — 95%CI 3.94–125.15; p=0.004). We also found that patients living with DM for more than 20 years were three times more likely to be impaired compared to those who were recently diagnosed with DM (aOR=3.63; 95%CI 1.70–18.81; p=0.026).

**Conclusion:**

This study revealed that cognitive impairment was relatively high in Bukavu city. It emphasizes the lack of effective tools to assess cognitive function. This requires, therefore, that research be adapted to the intellect and cultural experiences of the patients.

## RESUMO

A demência é uma questão de saúde pública global, afetando 57,5 milhões de pessoas com pelo menos um tipo de demência em 2019 em todo o mundo, com uma previsão de aumento para 152 milhões até 2050.

**Objetivo:**

Avaliou-se a função cognitiva em pacientes diabéticos com 60 anos ou mais na cidade de Bukavu, no leste da República Democrática do Congo (RDC).

**Métodos:**

Este estudo de caso-controle incluiu 123 pacientes com diabetes mellitus (DM) estabelecido e 123 controles com mais de 60 anos, com altas taxas de analfabetismo. A função cognitiva foi avaliada utilizando a versão swahili do Instrumento de Triagem Comunitária para Demência (*Community Screening Instrument for Dementia* — CSI-D).

**Resultados:**

O presente estudo revelou diferenças relacionadas à linguagem entre o swahili falado em outros países do leste da África, como Tanzânia e Quênia, onde o CSI-D swahili é prontamente aplicado, em comparação com o swahili falado em Bukavu (RDC). Observou-se também deficiência cognitiva em 18,7% dos 246 participantes. Notadamente, a taxa de prevalência de deficiência cognitiva foi maior no grupo não diabético (12,2 *versus* 25,2%; p=0,009). Participantes com 80 anos ou mais tiveram maior probabilidade de apresentar deficiência cognitiva em comparação com aqueles com menos de 80 anos (*odds ratios* ajustados — aOR=70,27; intervalo de confiança de 95% — IC95% 3,94–125,15; p=0,004). Também observou-se que pacientes vivendo com DM por mais de 20 anos tinham três vezes mais chances de serem afetados em comparação com aqueles que foram recentemente diagnosticados com DM (aOR=3,63; IC95% 1,70–18,81; p=0,026).

**Conclusão:**

Este estudo revelou que a deficiência cognitiva era relativamente alta na cidade de Bukavu. Enfatizou-se a falta de ferramentas eficazes para avaliar a função cognitiva, o que requer, portanto, que a pesquisa seja adaptada ao intelecto e às experiências culturais dos pacientes.

## INTRODUCTION

Dementia is a global public health issue, with 57.5 million people living with at least one type of it in 2019 worldwide, a figure projected to rise to 152 million by 2050^1^. The majority of people with dementia currently live in low- and middle-income countries (LMICs). Whereas dementia is highly prevalent in developed countries with current trends of stabilizing or even decreasing prevalence, its prevalence in African settings (Sub-Saharan African [SSA] regions, North and East Africa) is rising at the fastest rates. This prevalence ranges between 6.4 and 3.3% among people aged 65 and over living in Bangui and Brazzaville^2,3^.

Despite the paucity of data, the epidemiological trends show that the prevalence and incidence of diabetes mellitus (DM), which are currently estimated at 4.8% and 19.1 million in SSA, will be 5.7% and 41.4 million in 2035, respectively. This represents an increase of 109% compared to the 55% increase observed in other parts of the world. These estimates indicate the future importance of DM as well as its related dementia as serious health issues in SSA, which is undergoing a major demographic transition^4^.

Diabetes is an important risk factor for dementia^5-7^, predicting to affect 4.4% of the global population by 2030, compared to its incidence in 2000^8^. Recent evidence suggests that the increase in lifespan of the global population is associated with the increasing rate of cognitive impairment secondary to DM, given that age is a well-established risk factor for both cognitive impairment and DM^8,9^.

An estimated 5 to 15% of dementias are directly associated with DM, raising a global public health concern^10^. Studies suggest that the risk of dementia would be doubled as a result of an upsurge in dementia cases^10^, compared to those with severe hypoglycemia, who have a triple risk. Given the link between hypoglycemia and dementia, the latter should be considered one of the potential consequences of diabetes and highlights the need for dementia screening in older people with diabetes^11,12^.

To date, studies on dementia and diabetes are almost nonexistent in the Democratic Republic of the Congo (DRC). A recent preliminary study from the Neuropsychopathological Center of the University of Kinshasa (2023) showed that 6.2% of people living in the city of Kinshasa, the capital of the DRC, aged over 65 could have dementia^13^. A study carried out in Bukavu City (2012) estimated the prevalence of diabetes at 3.5%, suggesting this region to be one of the foci in SSA^14^. These figures are likely to be underestimated due to the limited awareness of metabolic diseases such as DM in the population of the city of Bukavu^15^.

It is clear that dementia and DM are increasingly prevalent and interrelated issues^16^. Several challenges could affect the provision of needed healthcare to people living with DM and dementia in the DRC, such as the lack of trained health workers (neurologists, neuropsychologists, etc.), limited research funding, and a lack of dementia screening tools appropriate to the DRC cultural context^17^.

In order to address the research and knowledge gap on dementia and diabetes in the DRC, this case-control study aimed to assess diabetes-related cognitive impairment and dementia in individuals aged 60 or over attending Panzi General Reference Hospital, Bukavu, in the DRC.

## METHODS

### Study setting

This case-control study involved participants recruited among diabetic patients attending the healthcare services at the internal medicine department of the General Reference Hospital of Panzi, in the commune of Ibanda, Bukavu, in the DRC. The General Reference Hospital of Panzi is a tertiary teaching hospital for the Faculty of Medicine of the Evangelical University in Africa. The department of internal medicine has 78 beds, with nearly ten hospital admissions and 30 outpatient consultations per day, and a team of 21 doctors and ten nurses. This health facility has implemented free monitoring and treatment for people living with diabetes mellitus and serves 50 people living with diabetes per month. Around 20 elderly diabetics are monitored monthly in this department. These individuals have or do not have comorbid hypertension and other vascular risk factors.

Three hundred patients with DM types 1 and 2 benefited from a follow-up after being diagnosed, based on blood sugar levels ≥126 mg/dL (≥7 mmol/L), twice or occasional blood sugar ≥200 mg/dL (≥11.1 mmol/L) over the age of 60 years.

### Study participants

**Figure 1:**
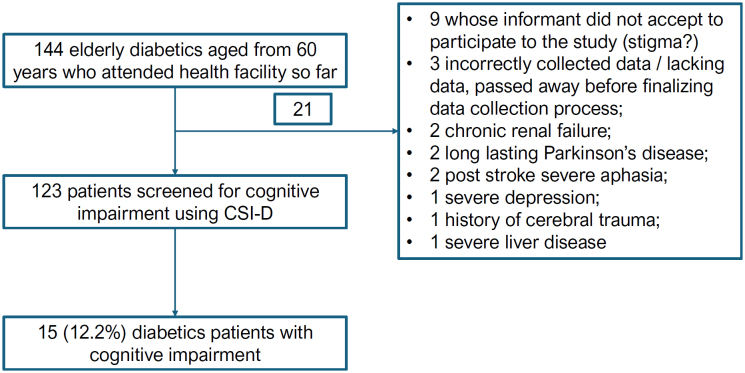
Flowchart showing study sampling of diabetic patients aged 60 years or older.

From February 2022 to June 2022, we enrolled 123 diabetic patients who attended the diabetic outpatient department at the General Reference Hospital of Panzi for follow-up and refill of their medication. Cases were patients who were previously diagnosed with DM types 1 or 2 and were aged 60 years or older. Controls were the elderly participants (aged from 60 years) admitted the same day and diagnosed with other conditions rather than DM or who were two years old or younger than the cases. This has been done in order to prevent a large age difference between cases and controls. We excluded:

- patients with neurological degenerative conditions, such as frontotemporal dementia;
- patients with schizophrenia, severe depression, and Parkinson disease;
- patients with obvious aphasia; and
- patients suffering from severe liver and renal disorders (Figure 1).

### Measurements

#### Demographic and clinical characteristics

We collected demographic data (sex, age, marital status, and education level) and information on risk factors for cardiovascular and cerebrovascular diseases (hypertension, history of stroke, total cholesterol, triglyceride, low-density lipoprotein, and glycated hemoglobin [HbA1c]) and full blood count. Peripheral blood samples were taken by automatic biochemical and hematology analyzers (semi-automatic spectrophotometer [BTS-350, Biosystems, Spain] and auto-hematology analyzer [MC-3200, MEDMAY, China]).

Patients with hypertension were defined as those who had a history of hypertension and were currently taking antihypertensive medications. According to the patient's self-reported medical history or the patient's current medical records, the course of DM spanned from the moment the patient was first diagnosed with diabetes until the time of this study. The age difference between patient and control might be two years.

#### Cognitive assessment

During the design of this study, one of the challenges was the choice of a dementia screening tool. Several tools assessing cognitive functions used in different studies are not adapted for use in many African settings, including the DRC, among older people with lower education, particularly in rural areas^18-26^.

Information is lacking on the appropriate measures for dementia and cognitive impairment screening in Swahili language speakers living in conflict zones in the eastern DRC. In order to overcome challenges due to the high rate of illiteracy among diabetic patients attending the General Reference Hospital of Panzi, we used the Community Screening Instrument for Dementia (CSI-D) to assess the cognitive functions^27,28^. Unlike several widely used instruments to assess cognitive function in various settings, the CSI-D assesses language, memory, orientation in time and space, praxis, and executive functions through 50 items. It has been used in research and clinical contexts in SSA, where there is not yet a standardized tool for the evaluation of cognitive disorders. The CSI-D is also designed for LMIC settings and those with low education. To also overcome the challenges of language, we selected the Swahili translation of CSI-D already used in epidemiological studies in Kenya and Tanzania^29,30^. Our previous studies have established cross-cultural methods to screen for age-related dementias and susceptibility genes such as apolipoprotein E. The CSI-D has also been translated into Kikuyu, a major language in Kenya, to evaluate dementia of the Alzheimer type. Using two sets of coefficients of cognitive and informant scores, the specificities of the discriminant function scores were remarkably similar (94%) in the Kenyan^28^ compared to the previous Ibadan sample^27^. We had proposed that the adapted CSI-D could be used to detect cognitive impairment or dementia among East Africans exposed to various vascular risk factors, as the main challenge is the lack of culturally adapted versions of the CSI-D in the DRC context. As a result, we offer a modified version of the DRC Swahili CSI-D (Supplementary Material - https://www.demneuropsy.com.br/wp-content/uploads/2024/06/DN-2023.0082-Supplementary-Material.docx).

Singling out specific items in the CSI-D, we found a particular problem in understanding items dealing with orientation. For instance, items 22 and 23 bearing the terms "district" and "village" found in the Tanzanian Swahili translation are now translated into "commune" and "neighborhood," respectively, in the section evaluating orientation.

**Figure 2:**
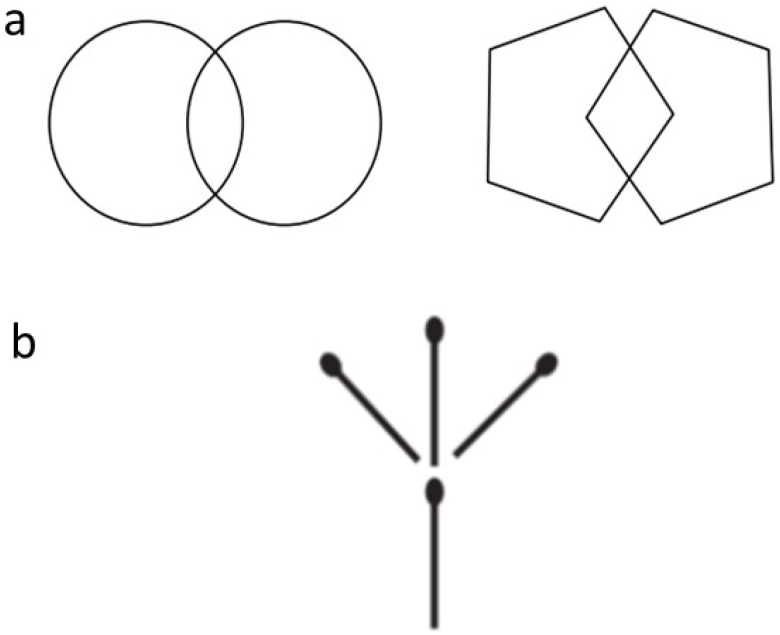
Praxis is assessed by the reproduction of the geometric diagrams (A) circles and pentagons. This can be challenging to many elderly subjects when they have not put pen to paper routinely. (B), replacement of the geometric diagrams with the stick design test. The subject is asked to reproduce the matchstick design in same manner, specifically in such a way that the heads of the matchsticks all point the same way.

As far as the evaluation of praxis was concerned, we have now replaced this test with the matchstick design to assess visuoconstructional ability and spatial orientation (Figure 2). The stick test was originally developed in Ibadan^31^ and asks subject to make the design using four matchsticks. The subject is shown once and then they have to copy exactly the design. The final score is formulated after a score of 1 is determined for each part of the design that is performed correctly. Such modifications are especially important in low literacy settings and where elderly persons may seldom have put pen to paper^32^.

**Figure 3:**
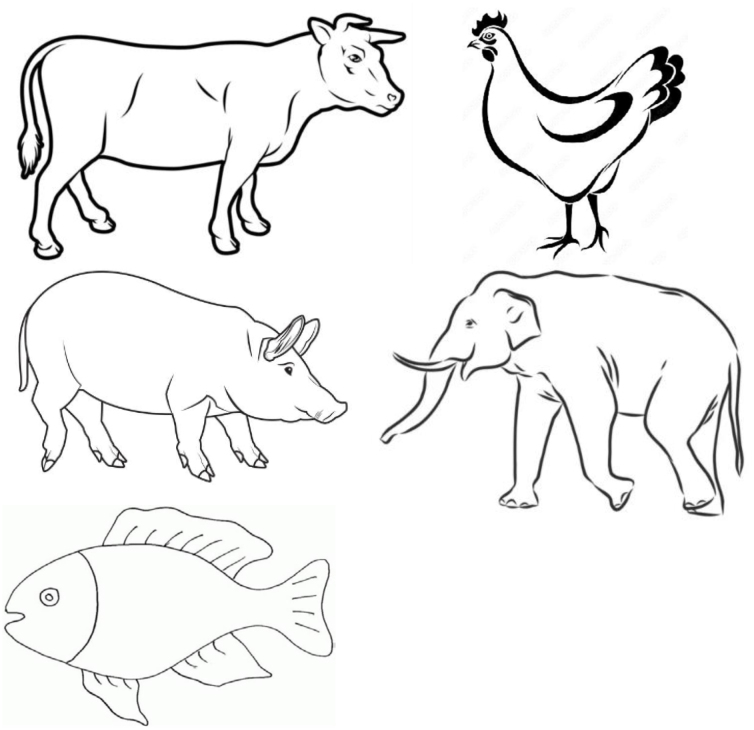
Naming objects and animals that are familiar to the participant for frontal lobe function. The Democratic Republic of the Congo Swahili version of the Community Screening Instrument for Dementia now uses these five animals, cow (*ngombe*), hen (*kuku*), pig (*nguruwe*), elephant (*tembo*) and fish (*samaki*), readily understood by those who are cognitively intact.

Regarding the evaluation of functions related to the frontal lobe, which consists of naming the images presented to the patients, we have now chosen images representing animals that are likely to be easily recognized by the patients in the particular locality. These include the cow (*ngombe)*, hen (*kuku*), pig (*nguruwe*), elephant (*tembo*), and fish (*samaki*) (Figure 3). This was an important substitution, as the use of certain objects, including animals, can vary remarkably even within the eastern African regions, where Swahili is spoken and used daily.

In order to avoid stigma, explanations adapted to each patient and their caregivers are often necessary to gain acceptance of the administration of the screening tool. To overcome the challenge of determining the cutoff for cognitive impairment for the CSI-D in this DRC context, given the lack of previous validation data, we referred to other African countries. As an initial solution, we elected to use the average CSI-D score reported in the recent study from Brazzaville: 25.5^2^ (Table 1)^2,13,33^. We expect that adjusting these norms will enable us to appropriately evaluate cognition and overcome stigma issues.

**Table 1:** Cutoffs used in recent African studies using Community Screening Instrument for Dementia for cognitive screening.

Study	Used cutoff	Inclusion age	Commentary
Ikanga et al.^13^	<25.5	≥65 years	Study conducted in Kinshasa, DRC
Kabamba et al.^33^	<25	≥18 years	Study conducted in Konzo-affected area (Kahemba City, Kwango province), DRC
Guerchet et al.^2^	<25.5	≥65 years	Study conducted in Bangui (Central African Republic) and Brazzaville (Republic of Congo)

Abbreviations: DRC, Republic of the Congo.

Furthermore, given that this study also sought to determine whether the CSI-D could be used to detect cognitive impairment in diabetics aged 60 years and older in an area where there is not sufficient health literacy to easily understand what cognitive impairment is (25.6% had never been to school, 25.6% had only primary level education), this was made possible by using the CSI-D in Swahili with modifications as discussed at the recent Symposium on Dementia and Brain Aging in LMICs in Nairobi^34^.

#### Procedures

Two research assistants purposely collected data through consecutive recruitment and face-to-face interviews. Each interview took about 45 minutes to one hour. Patients (cases and controls) seeking care at the aforementioned health facilities who expressed willingness to participate were asked to provide written informed consent after receiving details about the study from the research assistants. After giving consent, participants were administered a questionnaire by trained and fluent research assistants, and data were collected anonymously. The acceptance or denial to participate did not affect the access to care by the participant.

#### Statistical analysis

The data were encoded using Microsoft Excel 2016 and analyzed using the Stata SE 14.0 software (Stata Corp., LP, College Station, Texas, USA). Data from categorical variables were summarized using proportions and percentages, and from numerical variables using the mean with a standard deviation if normally distributed and the median with an interquartile range if not normally distributed. To compare proportions, Pearson's chi-square test was used (or Fisher's exact test for proportions less than or equal to 5). Student's *t* test was used to compare the means of biological parameters in patients with and without cognitive impairment. As far as the relationships between factors associated with cognitive impairment were concerned, univariable and multivariable logistic regression models were used. Variables that were statistically significant in bivariate analysis with a p-value of <0.2 were included in multivariate analyses using a regression model. The adjusted odds ratio (aOR) and the 95% confidence intervals (95% CI) were derived to measure the strength of the association between the variables. The significance threshold was set at p=0.05.

#### Ethical considerations

This study was conducted in accordance with the Declaration of Helsinki. Study approval was granted by the Institutional Review Board of the Provincial Health Division of South-Kivu (Reg. Number: CNES 001/DPSK/166PP/2021). All participants were informed about the purpose of the study. Written informed consent was obtained from all of them, or from informants in the case of participants with severe cognitive impairment. In order to propose a research tool adapted to the patients, we first trialed a dozen patients to identify the difficulties that should help us make this tool easy to administer in our context.

## RESULTS

### Cases and controls baseline characteristics

Among the 144 diabetic patients who attended the diabetic outpatient department at the General Reference Hospital of Panzi for follow-up and refill of their medication during the study period, only 123 were enrolled in this study. The main criteria for exclusion were: presenting conditions that can seriously affect neurocognitive function; or not being interested in participating in the study (Figure 1). These 123 diabetics were compared to 123 controls (aged from 60 years), admitted the same day, diagnosed with other conditions rather than DM, and being two years old or younger than the cases.

The majority of diabetics were male in the two groups (53.3; 64.2%), with 72% under the age of 70 in the diabetic patients group and 70.7% in the non-diabetic patients group. Most diabetic patients had low level of education (24.6% had never been to school, 25.6% had only primary level education, <7 years) (Table 2).

**Table 2:** Baseline characteristics of the cases and controls (non-diabetic).

Parameters		Total n=246 (%)	Cases n=123 (%)	Controls n=123 (%)	p-value
Sex					
	Male	131 (53.3)	52 (42.3)	79 (64.2)	<0.001*
	Female	115 (46.7)	71 (57.7)	44 (35.8)	
Age groups (years)					
	60–64	87 (35.4)	49 (39.8)	38 (30.9)	0.581*
	65–69	90 (36.6)	41 (33.3)	49 (39.8)	
	70–74	41 (16.7)	18 (14.6)	23 (18.7)	
	75–79	17 (6.9)	9 (7.3)	8 (6.5)	
	≥80	11 (4.5)	6 (4.9)	5 (4.1)	
Mean±SD		67.12±5.80	67.13±6.16	67.12±5.44	0.989†
Marital status					
	Married	178 (72.4)	89 (72.4)	89 (72.4)	0.655*
	Widower/widow	56 (22.8)	26 (21.1)	30 (24.4)	
	Divorced	9 (3.7)	6 (4.9)	3 (2.4)	
	Single	3 (1.2)	2 (1.6)	1 (0.8)	
Education					
	None	63 (25.6)	21 (17.1)	42 (34.1)	<0.001*
	Primary	63 (25.6)	43 (35.0)	20 (16.3)	
	Secondary	96 (39.0)	43 (35.0)	53 (43.1)	
	Tertiary	24 (9.8)	16 (13.0)	8 (6.5)	

Abbreviation: SD, standard deviation.

Notes:

* χ^2^ test;

† *t*-test.

### Cognitive impairment among cases and controls

The prevalence of cognitive impairment among the total participants was 18.7%. This prevalence of cognitive impairment was higher in non-diabetic patient groups (12.2 versus 25.2%; p=0.009). Cognitive impairment was mostly found in males (p=0.033). The older the patient was, the greater was the risk for him or her to suffer from cognitive impairment (72.58±5.62 years versus 65.87±5.08 years; p<0.001). DM that lasted longer (>20 years) was associated with a higher risk of presenting cognitive impairment (p=0.019). Cognitive impairment was also associated with a history of stroke (p = 0.004) and hypertension as a comorbidity (p=0.007) (Table 3).

**Table 3:** Cognitive impairment compared to baseline characteristics.

Variables		Total n=246 (%)		Cognitive impairment		p-value
				Yes n=46 (18.7%)	No n=200 (81.3%)	
Sex						
	Male	131 (53.3)	31 (67.4)		100 (50.0)	0.033*
	Female	115 (46,7)	15 (32,6)		100 (50,0)	
Age groups (years)						
	60–64	87 (35.4)	4 (8.7)		83 (41.5)	<0.001*
	65–69	90 (36.6)	6 (13.0)		84 (42.0)	
	70–74	41 (16.7)	20 (43.5)		21 (10.5)	
	75–79	17 (6.9)	9 (19.6)		8 (4.0)	
	≥80	11 (4.5)	7 (15.2)		4 (2.0)	
Associated clinical conditions						
	Diabetes only	53 (21.5)	2 (4.3)		51 (25.5)	0.007*
	Diabetes + hypertension	52 (21.1)	11 (23.9)		41 (20.5)	
	Others	141 (57.3)	33 (71.7)		108 (54.0)	
		Total n=123(%)	Diabetic patients			
			n=15 (12.2)		n=108 (87.8)	
Diabetes type						
	Type 1	3 (2.4)	0 (0.0)		3 (2.8)	0.675*
	Type 2	120 (97.6)	15 (100.0)		105 (97.2)	
History of stroke						
	Yes	7 (5.7)	4 (26.7)		3 (2.8)	0.004*
	No	116 (94.3)	11 (73.3)		105 (97.2)	
DM course						
	<10 years	90 (73.2)	9 (60.0)		81 (75.0)	0.019*
	10–20 years	23 (18.7)	2 (13.3)		21 (19.4)	
	>20 years	10 (8.1)	4 (26.7)		6 (5.6)	

Abbreviation: DM, diabetes mellitus.

Note:

* χ^2^ test.

Further analysis showed that it was more likely for a patient aged 80 or older to present with cognitive impairment than for those aged less than 80 in both groups. (aOR=70.27; 95%CI 3.94–125.15; p=0.004). Patients whose DM panned for more than 20 years were three times more likely to suffer from cognitive impairment compared to those whose DM had been recently diagnosed (aOR=3.63; 95%CI 1.70–18.81; p=0.026), and a history of stroke multiplied by 20 the risk of exhibiting cognitive impairment (aOR=20.05; 95%CI 2.12-188.9; p=0.009) (Table 4). There was no statistically significant relationship between HbA1c and cognitive disorders. HbA1c was even higher in cognitively impaired free patients (12.50±3.13 versus 15.35±5.38, p=0.048) (Table 5).

**Table 4:** Factors associated with cognitive impairment.

Variables		COR (95%CI)	p-value	aOR (95%CI)	p-value
Sex					
	Male	2.06 (1.05–4.06)	0.035*	2.83 (0.84–9.53)	0.092†
	Female	1 (reference)		1 (reference)	
Age groups (years)					
	60–64	1 (reference)		1 (reference)	
	65–69	1.48 (0.40–5.44)	0.553*	2.50 (0.35–17.94)	0.360†
	70–74	19.76 (6.10–64.01)	<0.001*	3.03 (0.39–23.61)	0.289†
	75–79	23.34 (5.88–93.10)	<0.001*	2.56 (0.23–27.62)	0.437†
	≥80	36.31 (7.43–177.37)	<0.001*	70.27 (3.94–125.15)	0.004†
DM course (years)					
	<10	1 (reference)		1 (reference)	
	10-20	0.85 (0.17–4.26)	0.851*	0.48 (0.88–2.65)	0.783†
	>20	6.01 (1.42–25.33)	0.015*	3.63 (1.70–18.81)	0.026†
Associated clinical conditions					
	Diabetes only	0.12 (0.02–0.55)	0.006*	0.24 (0.03–1.98)	0.189†
	Diabetes + hypertension	0.87 (0.40–1.89)	0.741*	1.71 (0.30–9.68)	0.541†
	Others	1 (reference)		1 (reference)	
Stroke history					
	No	1 (reference)		1 (reference)	
	Yes	12.72 (2.51–64.35)	0.002*	20.05 (2.12–188.99)	0.009†

Abbreviations: COR, crude odds ratios; aOR, ajusted odds ratios; DM, diabetes mellitus.

Notes:

* χ^2^ test;

† Logistic regression.

**Table 5:** Biological parameters in diabetics.

	Total (n=123)	Cognitive impairment		p-value
		Yes n=15	Non n=108	
	Mean±SD	Mean±SD	Mean±SD	
RC (/mm^3^)	4529297±417974	31333±212833	4584570±475186	0.724*
Hb (g/dL)	13.87±1.14	12.10±0.44	14.11±1.30	0.568*
VGM (fL)	92.43±57.95	87.59±6.02	93.10±61.81	0.731*
Hct (%)	39.20±7.02	39.20±5.80	39.20±7.19	0.999*
WBC (/mm^3^)	8846±5171	7885±4294	8980±5285	0.444*
Platelets (/mm^3^)	269146± 121792	287400±103327	266611±124349	0.537*
CRP (mg/L)	25.04±22.98	28.66±24.17	24.54±22.88	0.517 *
Cholesterol T (mmol/L)	4.79±1.68	5.03±1.31	4.76±1.73	0.564*
HDLc (mmol/L)	0.81±0.29	0.86±0.28	0.80±0.29	0.435*
LDLc (mmol/L)	3.12±1.71	3.34±1.28	3.08±1.76	0.598*
Triglycerides (mmol/L)	2.49±2.32	1.93±1.30	2.57± 2.43	0.317*
Glycemia (mmol/L)	11.60±7.91	8.80±4.74	12.25±8.35	0.007*
HbA1c (%)	15.00±5.24	12.50±3.13	15.35±5.38	0.048*
ALAT (IU/L)	17.83±10.70	20.10±15.12	17.52±9.99	0.383*
ASAT (IU/L)	19.1±11.1	17.01±6.68	19.42±11.62	0.434*
Gamma GT (IU/L)	26.16±15.60	24.72±8.85	26.36±16.34	0.704*
Urea (mmol/L)	9.03±7.07	10.18±6.71	8.87±7.13	0.502*
Creatinine (μmol/L)	242.80±256.40	238.75±216.15	243.37±262.38	0.948*
GFR (mL/min/1.73 m²)	46.76±26.95	43.65±20.01	47.19±27.82	0.634*

Abbreviations: RC, red cells; HbA1c, glycated hemoglobin; ALAT, alanine transaminase; ASAT, aspartate transaminase; Gamma-GT, Gamma-glutamyltransferase; GFR, Glomerular filtration rate; SD, standard deviation.

Note:

* *t*-test; Bold indicates statistically significant p-values.

## DISCUSSION

After explanations on how the study was conducted and the expected results, 6% of diabetics refused to participate in the research (Figure 1). It is known that a common problem in LMICs for people living with dementia is dealing with stigmatization^3^. As this cultural or supernatural stigmatization is rooted in belief systems^35,36^, cognitively impaired patients are thought to be witches. This may help explain this high refusal rate. The patient and caregivers feared that they would be stigmatized if the interview revealed difficulties in answering the different CSI-D items.

Our study revealed that the prevalence of cognitive impairment was 18.7% in 60-year-old or older participants. The prevalence of cognitive impairment was higher in non-diabetic patient groups, likely because of other conditions that affect cognition. In this study, cognitive impairment was predominant in males (p=0.033). Human gender differences in cognitive function have been reported, with studies generally concluding that women have better cognitive function and appear to be cognitively >10 years younger than men at the age of 65^37^. Most sex differences in cognitive function seem to narrow with aging. Some arguments have been made that women decline faster in memory and information processing speed, which may reflect the sex differences in dementia prevalence as observed in old age. This issue remains controversial^38^. The older the patient was, the greater was the risk for him or her to suffer from cognitive impairment. DM that lasted longer (>20 years) was associated with a higher risk of presenting cognitive impairment. Cognitive impairment was also associated with a history of stroke, and hypertension as a comorbidity.

This prevalence is consistent with the impairment reported in Sub-Saharan Africans. The numbers vary per country, ranging from 6.3 to 25% (95%CI 21.2–29.0)^39^. Recently, it has been shown that, among other characteristics, cerebral infarction, the duration of diabetes, and HbA1c were associated with the incidence of cognitive impairment in patients suffering from diabetes^40,41^. Hypertension has been identified as a potentially modifiable factor for cognitive impairment^42^. Furthermore, cognitive impairment appears to be an age-dependent clinical condition, as its prevalence is described to be elevated in the population aged 80 years or older in the Chinese population^43^.

It has been reported that between 15 and 30% of stroke survivors have a permanent handicap, including physical, social, and cognitive functions. Of people who survive an ischemic stroke, 25–30% go on to acquire immediate or delayed vascular cognitive impairment or vascular dementia. The following variables may influence the cognitive status of stroke survivors: age, education level, history of stroke, prior transient ischemic attack, DM, hypertension, types of stroke, vascular comorbidities, affected area, size and location of infarction, depressive symptoms, genetic variants, and physical function^44^.

There is a link between hypertension and the risk of cognitive dysfunction. Chronic cerebral hypoperfusion is a result of structural arterial wall changes linked to untreated long-term hypertension^45^. It is thought that the age of onset of hypertension, the chronicity of hypertension, and the antihypertensive medication used are important factors in determining the risk of cognitive impairment^46^.

Unsurprisingly, a history of stroke^47^, the course of DM, being elderly (≥80 years), and hypertension are associated with the cognitive impairment except HbA1c in this study. As far as HbA1c is concerned, this may be due to the study population's limited size, as the relationship between HbA1c and cognitive impairment appears not to be linear^48^. It's important to mention that the Bruce D.G. et al. study revealed no significant differences in fasting glucose and HbA1c in diabetes patients with or without severe cognitive impairment^49^.

### Feasibility testing of the Community Screening Instrument for Dementia and subsequent cultural modification

In the feasibility testing phase of the study, we were confronted with several difficulties related to the language, such as the fact that the Swahili language spoken in Tanzania or Kenya is not the same as that spoken in the city of Bukavu. Another major challenge was the evaluation of praxis, particularly copying of geometric diagrams such as circles and pentagons (Figure 2), which posed a comprehension problem for the less literate, especially for the 12 CSI-D in the identified reports.

### Stigma and engagement

Another difficulty encountered is the fear experienced by the caregivers of diabetic patients enrolled in our study. While these changes were discussed at the recent Symposium on Dementia and Brain Aging in Nairobi^34^, we readily discerned that there is a cultural influence on how the communities perceive cognitive disorders. As elsewhere in SSA, many believe that they are a negative effect of witchcraft. This leads to an increased refusal rate for participation in studies such as ours (Figure 1). These common beliefs in the community may lead participants to experience stigma and popular condemnation.

Thus, a significant challenge in the case of our study was to determine the cutoff for cognitive impairment for the CSI-D in this DRC context, also given the lack of previous validation data.

### Strengths and weakness of the study

This cross-sectional case-control study involved participants recruited among diabetic patients attending the healthcare services at the internal medicine department of the tertiary hospital and not in the general population. First, it is tricky to address cognitive impairment causality by using a cross-sectional study^50^. Secondly, having a multi-center approach could be a source of variability. Despite these limitations, this is the first case-control study to screen for neurocognitive disorders in older adults treated for DM in the Democratic Republic of the Congo. This attempt required researchers to adapt the research tool (CSI-D) to the intellectual and cultural experiences of the patients or participants living in Bukavu city. This was made possible by using the CSI-D in Swahili with modifications as discussed at the recent Symposium on Dementia and Brain Aging in LMICs in Nairobi^34^: matchstick design to assess visuoconstructional ability and spatial orientation and images representing animals that are likely to be easily recognized by the patients in the particular locality to assess functions related to the frontal lobe, and that consists in naming the images presented to the patients. This study has therefore provided a reference tool for further research to be conducted in areas where patients with similar sociodemographic characteristics are present. Finally, comparing diabetics to a control group has helped to strengthen the validity of our results.

In conclusion, cognitive impairment was relatively high in our study. Our results also emphasize the lack of effective tools to assess cognitive function in DRC and similar settings when the tools are translated into local dialects. Therefore, it is important to create culture-specific tools for different localities in SSA settings. Screening for neurocognitive disorders in older adults treated for chronic metabolic disease in the city of Bukavu requires researchers to adapt their research to the intellect and cultural experiences of the patients or participants. We propose modified versions of the CSI-D that can be used and implemented by investigators when assessing cognitive function in community subjects living in regions and communities where even the same language may be spoken. Advocacy in favor of a screening tool for cognitive disorders adapted to SSA conditions is necessary.

## References

[1] GBD 2019 Dementia Forecasting Collaborators (2022). Estimation of the global prevalence of dementia in 2019 and forecasted prevalence in 2050: an analysis for the Global Burden of Disease Study 2019. Lancet Public Health.

[2] Guerchet M, M’Belesso P, Mouanga AM, Bandzouzi B, Houinato D, Paraïso M (2009). Prévalence et facteurs de risque des troubles cognitifs et démence chez les personnes âgées vivant en Afrique Centrale: Bangui & Brazzaville. Rev Neurol (Paris).

[3] Akinyemi RO, Yaria J, Ojagbemi A, Guerchet M, Okubadejo N, Njamnshi AK (2022). Dementia in Africa: current evidence, knowledge gaps, and future directions. Alzheimers Dement.

[4] Kalaria RN, Maestre GE, Arizaga R, Friedland RP, Galasko D, Hall K (2008). Alzheimer's disease and vascular dementia in developing countries: prevalence, management, and risk factors. Lancet Neurol.

[5] Cheng G, Huang C, Deng H, Wang H (2012). Diabetes as a risk factor for dementia and mild cognitive impairment: a meta-analysis of longitudinal studies. Intern Med J.

[6] Gudala K, Bansal D, Schifano F, Bhansali A (2013). Diabetes mellitus and risk of dementia: a meta-analysis of prospective observational studies. J Diabetes Investig.

[7] Li J, Shao YH, Gong YP, Lu YH, Liu Y, Li CL (2014). Diabetes mellitus and dementia – a systematic review and meta-analysis. Eur Rev Med Pharmacol Sci.

[8] Wild S, Roglic G, Green A, Sicree R, King H (2004). Global prevalence of diabetes: estimates for the year 2000 and projections for 2030. Diabetes Care.

[9] Prince M, Bryce R, Albanese E, Wimo A, Ribeiro W, Ferri CP (2013). The global prevalence of dementia: a systematic review and metaanalysis. Alzheimers Dement.

[10] Kloppenborg RP, van den Berg E, Kappelle LJ, Biessels GJ (2008). Diabetes and other vascular risk factors for dementia: which factor matters most? A systematic review. Eur J Pharmacol.

[11] Nguyen S, Major K, Démonet JF, Smith C, Rubli E, Humbert M (2014). Diabetes and dementia: the dangerous liaisons?. Rev Med Suisse.

[12] Bellia C, Lombardo M, Meloni M, Della-Morte D, Bellia A, Lauro D (2022). Diabetes and cognitive decline. Adv Clin Chem.

[13] Ikanga J, Reyes A, Kaba D, Akilimali P, Mampunza S, Epenge E (2023). Prevalence of suspected dementia in a sample of adults living in Kinshasa-Democratic Republic of the Congo. Alzheimers Dement.

[14] Katchunga P, Masumbuko B, Belma M, Kashongwe Munogolo Z, Hermans MP, M'buyamba-Kabangu JR (2012). Age and living in an urban environment are major determinants of diabetes among South Kivu Congolese adults. Diabetes Metab.

[15] Katchunga PB, Malanda B, Mweze MC, Dupont B, M’Buyamba-Kabangu JR, Kashongwe Z (2012). Knowledge of the general population about hypertension and diabetes mellitus in South Kivu, Democratic Republic of Congo]. Rev Epidemiol Sante Publique.

[16] Biessels GJ, Despa F (2018). Cognitive decline and dementia in diabetes mellitus: mechanisms and clinical implications. Nat Rev Endocrinol.

[17] Onohuean H, Akiyode AO, Akiyode O, Igbinoba SI, Alagbonsi AI (2022). Epidemiology of neurodegenerative diseases in the East African region: a meta-analysis. Front Neurol.

[18] Folstein MF, Folstein SE, McHugh PR (1975). "Mini-mental state". A practical method for grading the cognitive state of patients for the clinician. J Psychiatr Res.

[19] Borson S, Scanlan J, Brush M, Vitaliano P, Dokmak A (2000). The mini-cog: a cognitive "vital signs" measure for dementia screening in multi-lingual elderly. Int J Geriatr Psychiatry.

[20] Nasreddine ZS, Phillips NA, Bédirian V, Charbonneau S, Whitehead V, Collin I (2005). The Montreal Cognitive Assessment, MoCA: a brief screening tool for mild cognitive impairment. J Am Geriatr Soc.

[21] Miller JM, Pliskin NH (2006). The clinical utility of the Mattis Dementia Rating Scale in assessing cognitive decline in Alzheimer's disease. Int J Neurosci.

[22] Mioshi E, Dawson K, Mitchell J, Arnold R, Hodges JR (2006). The Addenbrooke's Cognitive Examination Revised (ACE-R): a brief cognitive test battery for dementia screening. Int J Geriatr Psychiatry.

[23] Rowland JT, Basic D, Storey JE, Conforti DA (2006). The Rowland Universal Dementia Assessment Scale (RUDAS) and the Folstein MMSE in a multicultural cohort of elderly persons. Int Psychogeriatr.

[24] Kiernan RJ, Mueller J, Langston JW, Van Dyke C (1987). The Neurobehavioral Cognitive Status Examination: a brief but quantitative approach to cognitive assessment. Ann Intern Med.

[25] LoGiudice D, Smith K, Thomas J, Lautenschlager NT, Almeida OP, Atkinson D (2006). Kimberley Indigenous Cognitive Assessment tool (KICA): development of a cognitive assessment tool for older indigenous Australians. Int Psychogeriatr.

[26] Welsh KA, Butters N, Mohs RC, Beekly D, Edland S, Fillenbaum G (1994). The Consortium to Establish a Registry for Alzheimer's Disease (CERAD). Part V. A normative study of the neuropsychological battery. Neurology.

[27] Hall KS, Gao S, Emsley CL, Ogunniyi AO, Morgan O, Hendrie HC (2000). Community screening interview for dementia (CSI ’D’); performance in five disparate study sites. Int J Geriatr Psychiatry.

[28] Chen CH, Mizuno T, Elston R, Kariuki MM, Hall K, Unverzagt F (2010). A comparative study to screen dementia and APOE genotypes in an ageing East African population. Neurobiol Aging.

[29] Longdon AR, Paddick SM, Kisoli A, Dotchin C, Gray WK, Dewhurst F (2013). The prevalence of dementia in rural Tanzania: a cross-sectional community-based study. Int J Geriatr Psychiatry.

[30] Paddick SM, Gray WK, McGuire J, Richardson J, Dotchin C, Walker RW (2017). Cognitive screening tools for identification of dementia in illiterate and low-educated older adults, a systematic review and meta-analysis. Int Psychogeriatr.

[31] Baiyewu O, Unverzagt FW, Lane KA, Gureje O, Ogunniyi A, Musick B (2005). The Stick Design test: a new measure of visuoconstructional ability. J Int Neuropsychol Soc.

[32] Kalaria RN (2003). Dementia comes of age in the developing world. Lancet.

[33] Kabamba VH, Luwa E-A DO, Mumba-Ngoyi D, Boivin MJ, Tshala-Katumbay D (2022). Facteurs de risque associés aux altérations neurocognitives observées chez l'adulte sous régime alimentaire principalement à base de manioc toxique. Revue de Neuropsychologie.

[34] Maestre G, Carrillo M, Kalaria R, Acosta D, Adams L, Adoukonou T (2023). The Nairobi Declaration-Reducing the burden of dementia in low- and middle-income countries (LMICs): Declaration of the 2022 Symposium on Dementia and Brain Aging in LMICs. Alzheimers Dement.

[35] Brooke J, Ojo O (2020). Contemporary views on dementia as witchcraft in sub-Saharan Africa: a systematic literature review. J Clin Nurs.

[36] Spittel S, Maier A, Kraus E (2019). Awareness challenges of mental health disorder and dementia facing stigmatisation and discrimination: a systematic literature review from Sub-Sahara Africa. J Glob Health.

[37] Nooyens ACJ, Wijnhoven HAH, Schaap LS, Sialino LD, Kok AAL, Visser M (2022). Sex differences in cognitive functioning with aging in the Netherlands. Gerontology.

[38] Caselli RJ, Dueck AC, Locke DEC, Baxter LC, Woodruff BK, Geda YE (2015). Sex-based memory advantages and cognitive aging: a challenge to the cognitive reserve construct?. J Int Neuropsychol Soc.

[39] Mavrodaris A, Powell J, Thorogood M (2013). Prevalences of dementia and cognitive impairment among older people in sub-Saharan Africa: a systematic review. Bull World Health Organ.

[40] Ma ZY, Wu YY, Cui HYL, Yao GY, Bian H (2022). Factors influencing post-stroke cognitive impairment in patients with type 2 diabetes mellitus. Clin Interv Aging.

[41] Ehtewish H, Arredouani A, El-Agnaf O (2022). Diagnostic, prognostic, and mechanistic biomarkers of diabetes mellitus-associated cognitive decline. Int J Mol Sci.

[42] Kivipelto M, Mangialasche F, Ngandu T (2018). Lifestyle interventions to prevent cognitive impairment, dementia and Alzheimer disease. Nat Rev Neurol.

[43] Xing X, Yang X, Chen J, Wang J, Zhang B, Zhao Y (2024). Multimorbidity, healthy lifestyle, and the risk of cognitive impairment in Chinese older adults: a longitudinal cohort study. BMC Public Health.

[44] Kalaria RN, Akinyemi R, Ihara M (2016). Stroke injury, cognitive impairment and vascular dementia. Biochim Biophys Acta.

[45] Borshchev YY, Uspensky YP, Galagudza MM (2019). Pathogenetic pathways of cognitive dysfunction and dementia in metabolic syndrome. Life Sci.

[46] Walker KA, Power MC, Gottesman RF (2017). Defining the relationship between hypertension, cognitive decline, and dementia: a review. Curr Hypertens Rep.

[47] El Husseini N, Katzan IL, Rost NS, Blake ML, Byun E, Pendlebury ST (2023). Cognitive impairment after ischemic and hemorrhagic stroke: a Scientific Statement from the American Heart Association/American Stroke Association. Stroke.

[48] Xu L, Xiong Q, Du Y, Huang LW, Yu M (2023). Nonlinear relationship between glycated hemoglobin and cognitive impairment after acute mild ischemic stroke. BMC Neurol.

[49] Bruce DG, Davis WA, Casey GP, Starkstein SE, Clarnette RM, Foster JK (2008). Predictors of cognitive impairment and dementia in older people with diabetes. Diabetologia.

[50] Savitz DA, Wellenius GA (2023). Can cross-sectional studies contribute to causal inference? It depends. Am J Epidemiol.

