# Supplementary material for: Challenges in evaluating cognitive impairment in diabetics in the Democratic Republic of the Congo
Source: Dement Neuropsychol. 2024 Sep 23;18:e20230082. doi: 10.1590/1980-5764-DN-2023-0082 (PMC11421554; doi:10.1590/1980-5764-DN-2023-0082)
Supplement: Supplementary file 1 [file 1980-5764-DN-18-e20230082-Suppl01.docx]

**The Community Screening Instrument for Dementia (CSI-D)- Swahili**

## A: Usahili wa mgonjwa/Patient Interview

**Kwanza ningependa kukuuliza maswali kuhusu masomo na kazi yako.**

I would like to begin by asking you about school and also about your work

1. **Wewe umesoma shule?** / Did you go to school?

**Hapana/**No 0 Ndio/Yes 1 [__]

1. **Unaweza kusoma na kuandika? (gazeti/barua)** / Can you read and write?

**Hapana/**No 0 Ndio/Yes 1 [__]

1. **Ulifikia kiwango gani shuleni?** / What was the highest grade you reached in school?

State answer________________

1. **Shuguli gani ulizokuwa nazo maishani mwako. Kama ni kufanya kazi, ulifanya kazi gani?**

/ What was your main occupation in life? What kind of job did you have?

4a) **Kazi maalum** / Primary Occupation ______________________________________________

4b) **Muda gani?** / How long did you do that work? Record number of years__________________

4c) **Kazi zingine** / Secondary occupation ______________________________________________

4d) **Muda?** / How long did you do that work? Record number of years______________________

1. **Ningependa ulikumbuke jina langu. Mimi ninaitwa (jina la ukoo – surname)____________Tafadhali litilie maanani hilo jina, kwa vile nitataka ulitaje baadaye.**

I’d like you to remember my name. My last name is ____________ Can you repeat that please? I want you to remember it because I will ask you my name a little later.

**Hawezi kurudia jina lako/**Cannot repeat name 0 **Anarudia jina vizure** Successfully repeats name 1 |___|

Language expression-Naming

**Tutaanza kwa kutaja vitu kwa hivyo nitakuonyesha kitu na kisha utakitaja. Kwa mfano,**

We will begin with naming things. I will point to something and I would like you to tell me the name of the object.

1. *(Interviewer shows a pencil/****Mwonyeshe penseli au kalamu)***

**Hii inaitwaje? /** What is this called? **Penseli or kalamu**

**Siyo sawa/**Incorrect 0 **Sawa/** Correct 1 |___|

1. *(Interviewer points to their watch/****Mwonyeshe saa ya mkonoi****)*

**Na hii?** / What is this? **Saa ya mkono**

**Siyo sawa/**Incorrect 0 **Sawa/** Correct 1 |___|

1. *(Interviewer pats chair****/Mwonyeshe kiti****)*

**Na hiki je?** / What about this? **Kiti**

**Siyo sawa/**Incorrect 0 **Sawa/** Correct 1 |___|

1. *(Interviewer points to shoes/****Mwonyeshe viatu****)*

**Na hivi? /** And these? **Viatu**

**Siyo sawa/**Incorrect 0 **Sawa/** Correct 1 |___|

1. *(Interviewer shows their fingers/****Mwonyeshe vidole****)*

**Hivi vinaitwaje? /** What do we call these? **Vidole**

**Siyo sawa/**Incorrect 0 **Sawa/** Correct 1 |___|

1. *(Interviewer points to their knee/****Mwonyeshe Goti****)*

**Tunaitaje hii?**/ What do we call this? **Goti**

**Siyo sawa/**Incorrect 0 **Sawa/** Correct 1 |___|

1. *(Interviewer points to their shoulder/****Mwonyeshe bega****)*

**Na hii sehemu ya mwili inaiwaje?** / What do we call this? **Bega**

**Siyo sawa/**Incorrect 0 **Sawa/** Correct 1 |___|

Language Expression – definition

**Nilikuwa nikikuonyesha vitu kisha ukivitaja majina. Sasa nitataja majina ya vitu Fulani kisha wewe unielezee ni nini?**

I was just showing you things and you told me what we call them. Now I will tell you the name of something and I want you to describe what it is. For example

1. **Daraja ni nini?** / What is a bridge?_________________________________________________________

**Siyo sawa/**Incorrect 0 **Sawa/** Correct 1 |___|

1. **Nyundo hutumika kwa kazi gani**?/ What do you do with a hammer?______________________________

**Siyo sawa/**Incorrect 0 **Sawa/** Correct 1 |___|

1. **Watu hufanya nini kanisani?** / What do people do in a church?__________________________________

**Siyo sawa/**Incorrect 0 **Sawa/** Correct 1 |___|

1. **Ni wapi watu hununua madawa?** / Where do we go to buy medicine?_____________________________

**Siyo sawa/**Incorrect 0 **Sawa/** Correct 1 |___|

Language Expression – Repetition

1. **Sasa ninataka urudie vile nitasema** / Now I would like you to repeat what I say

(Only one presentation is allowed, so the interviewer must read the phrase clearly and slowly enunciating carefully)

**Haraka haraka haina Baraka** / Hurry, hurry has no blessing

**Siyo sawa/**Incorrect 0 **Sawa/** Correct 1 |___|

Memory - Recall

1. **Unakumbuka Jina Langu? Litaje?** / Do you remember my name? What is it?

**Siyo sawa/**Incorrect 0 **Sawa/** Correct 1 |___|

**Ninaitwa __________________, Nitakuuliza tena hivi punde.**

1. **Haya tuingilie kitu kingine. Nitakupatia kikundi cha vitu, nawe unitajie vitu vilivyoko katika hiki kikundi upesi uwezavyo. Kwa mfano, ktika kikundi cha mavazi tuko na vitu vifuatavyo, shati, tai, kofia…..**

**Sasa nitajie vitu vilivyoko katika kikundi cha wanyama. Kwa dakika moja ninataka unitajie wanyama wote, wawe wa angani, ardhini, majini au msituni. Tayari, anza.**

Ask patient to state as many animals as possible in 1 minute (can be any kind of animals – in land, water, forest..)

*Number of animals/Idacli ya wanyama aliotaja ________* |___|___|

1. **Sasa nitayataja maneno matatu na ningependa uya kumbuke na uyarudie haya maneno. Nanaitwa kuuliza baadaye / Now I am going to tell you three words and I would like you to repeat them after me**

**Rudia haya maneno**. / Repeat after me these words

**Baisikeli** / bicycle **Nyumba** / House **Mkate** / bread

*Score one point for each correct word on first attempt/****Alama moja kwa kila neno sawa*** |___|

*20 a. Then go on to repeat the three words, up to a total of six times until the subject has remembered them all correctly- if subject unable to repeat words after 6 attempts, put 7.****/ Rudia haja maneno matatu hadi mara sita mpaka aweze kukumbuka vizuri kama hawezi kurudia baada ya kumwambia mara sita andika 7***

Record number of trials until repeated successfully/**Andika namba aliyojaribu hadi akaweza kutaja vizuri** |___|

**Jaribu uyakumbuke haya maneno kwa vile nitahitaji uyakumbuke baadaye.**

Very good, now try to remember these words because I will be asking you later

Orientation to place

**Sasa ningependa kukuuliza maswali kuhusu hapa kwenu.** / Now I would like to ask some questions about your home, this area.

1. **Huu ni mkoa gani?** / What is the name of this province/region? Record answer_________________(Bukavu)

**Siyo sawa/**Incorrect 0 **Sawa/** Correct 1 |___|

1. **Hii ni** **commune gani?**/ What is the name of this commune? Record answer___________________­(Bukavu)

**Siyo sawa/**Incorrect 0 **Sawa/** Correct 1 |___|

1. **Hiki ni kitongoji gani?**/ What is the name of this neighborhood? Record answer_________________________

**Siyo sawa/**Incorrect 0 **Sawa/** Correct 1 |___|

1. **Jirani yako anaitwa nani? /** Who lives next door/what is the name of your neighbor? Record answer______

**Siyo sawa/**Incorrect 0 **Sawa/** Correct 1 |___|

1. **Jina la mwenyekiti wa commune ni nini**? What is the name of the Village Chairman? Record answer_______

**Siyo sawa/**Incorrect 0 **Sawa/** Correct 1 |___|

1. **Barabara kubwa hapa inatoka wapi na kuelekea wapi?** / The large road near here, goes from where to where? Record answer______________

**Siyo sawa/**Incorrect 0 **Sawa/** Correct 1 |___|

1. **Soko kubwa hapa liko upande gani**? / Where is the large market for this place? Record answer_________

**Siyo sawa/**Incorrect 0 **Sawa/** Correct 1 |___|

1. **Soko kubwa hapa inaitwaje?** What is the name of that market? Record answer______________________

**Siyo sawa/**Incorrect 0 **Sawa/** Correct 1 |___|

Recall

1. **Unakumbuka yale meneno matatu niliyokutajia hapo awali (dakika chache zilizo pita)? Yataje** / Do you remember the three words I told you a few minutes ago?

**Baisikeli** / bicycle Incorrect 0 Correct 1

**Nyumba** / house Incorrect 0 Correct 1

**Mkate** / bread Incorrect 0 Correct 1 [___]

Orientation to time

**Hebu sasa nikuulize kuhusu wakati** / Now I would like to ask some questions about time.

1. **Huu ni mwezi gani?** / What month is it?

**Siyo sawa/**Incorrect 0 **Sawa/** Correct 1 |___|

1. **Leo ni siku gani?** / What day of the week?

**Siyo sawa/**Incorrect 0 **Sawa/** Correct 1 |___|

1. **Huu ni mwaka gani?** / What year is it?

**Siyo sawa/**Incorrect 0 **Sawa/** Correct 1 |___|

1. **Ni msimu (pembe) gani wa mwaka?** / What season is it? (Can state wet/dry)

**Siyo sawa/**Incorrect 0 **Sawa/** Correct 1 |___|

1. **Sasa hivi ni asubuhi, mchana au usiku?** / What part of the day is it? For example is it morning, afternoon or evening?

**Siyo sawa/**Incorrect 0 **Sawa/** Correct 1 |___|

1. **Kulinyesha jana?** / Did it rain yesterday?

**Siyo sawa/**Incorrect 0 **Sawa/** Correct 1 |___|

Language Comprehension – Motor Response

**Sasa ninakuomba ufanye vile nisemavyo. Ninataka usikilize kwa makini, kwa sababu nitakueleza mara moja tu.**

I am going to ask you to carry out some actions so please listen carefully, because I will only tell you one time

(Interviewer -, give complete instructions at one time, do not give step by step)

1. **Tafadhali tingisha kichwa chako.** / Please shake your head

**Siyo sawa/**Incorrect 0 **Sawa/** Correct 1 |___|

1. **Onyesha kwa mkono kwanza dirisha kisha mlango**. / Please point first to the window and then to the door

**Siyo sawa/**Incorrect 0 **Sawa/** Correct 1 |___|

1. **Nitakupa karatasi na ninaomba uichukuwe kwa mkono wa kulia, uikunje katikati kwa mikono yote miwili, kisha uweke kwenye mapaja yako.** / I’m going to give you a piece of paper. When I do, take the paper in your right hand, fold the paper in half with both hands, and put the paper down on your lap. (Max score 3)

**Siyo sawa/**Incorrect 0 Uses right hand 1 Folds in two 1 Places on lap 1 |___|

**Kumbukumbu**/Memory

1. **Sasa ninataka usikilize kwa makini nikikusomea hadithi fupi kisha nikimaliza ujaribu kurudia vile nilivyoisoma**. / Now I will tell a short story, then I will ask you to repeat as much of the story as you can remember. I want you to listen very carefully because I want you to try to tell me the whole story with as many details as you can remember.

**Watoto watatu walikuwa peke yao nyumbani na nyumba yao ikaanza kuteketea. Mtu mmoja shujaa alipandia dirishani na kuweza kuwaokoa. Wote walikuwa salama ingawaje walikuwa na majeraha madogo hapa na pale**./ Three children were alone at home and the house caught on fire. A brave man managed to climb in a back window and carry them to safety. Aside from minor cuts and bruises, all were well.

**Haya irudie sasa.** / Now I would like you tell me the story in as much detail as possible

**Watoto watatu** / 3 children 1

**Nyumba kuteketea** / house on fire 1

**Shujaa kupanda** / brave man climbed 1

**Watato kuokolewa** / children rescued 1

**Majeraha madogo madogo** / minor injuries 1

**Wote kuwa salama** / everyone well 1 (Max Score 6) |___|

Praxis – copying

**40/41. Je, unaweza kufanya maumbokama inavyoonekana hapa chini kwa kutumia njiti nne za kiberiti. Mimi nitakuonyesha mara moja na kisha wewe utarudia kama mimi nilifanywa) (Mtahini anapaswa kufanya maumbo ya kwanza kwa kutumia njiti za kiberiti na hasa kuelezea kwa ufasaha kuwa vichwa vyote vine vielekee upande mmoja. Mara mtahini anapomaliza kufanya umbo, kusanya njiti za kiberiti katika rundo na kuziweka. mbele ya mtu kuwa anayehojiwa, ni sawa mtu kuona umbo hili hapa chini wakati wakitengeneza um** / Can you make the design shown below using these four matchsticks? I will show you once and then you have to copy exactly. (The examiner should make the design first using the matchsticks and specifically point out to the person that the heads of the matchsticks all need to point the same way. Once the examiner has made the shape, collect up the matchsticks in a bunch and place them in front of the person being interviewed.)

#

#

**40.** Score/**Alama**: Incorrect/**Si Sahihi** 0 **41.** Score/**Alama**: Incorrect/**Si Sahihi** 0

Partially Correct/**Sahihi kidogo** 1 Partially Correct/**Sahihi kidogo** 1

Completely Correct/**Sahihi** 2 |___| Completely Correct/**Sahihi** 2 |___|

Long term memory

1. **Mwaka 1961 tukio gani muhimu katika historia ya Tanzania ilitokea?** / In 1961, what happened in Tanzania? **Jamuhuri / Huru** / Independence

**Siyo sawa/**Incorrect 0 **Sawa/** Correct 1 |___|

1. **Raisi wa kwanza wa nchi hii alikuwa nani?** / Who was the first president of this country?

**Siyo sawa/**Incorrect 0 **Sawa/** Correct 1 |___|

1. **Raisi wa sasa wa Tanzania ni nani?** / Who was the current president of this Tanzania?

**Siyo sawa/**Incorrect 0 **Sawa/** Correct 1 |___|

Additional frontal questions

1. **Je, unaweza kuniambiya kwa nini embe na chungwa zina fanana au kwanini zinamahusiano?** / Can you tell me why a mango and an orange are similar?

**Siyo sawa/**Incorrect 0 **Sawa/** Correct 1 |___|

1. **Unaweza kunielezea wapi unapoishi**? **Unaweza kunielezea kidogo kuhusu unapoishi?** /Can you tell me a sentence about where you live? (to include a verb and a noun)

**Siyo sawa/**Incorrect 0 **Sawa/** Correct 1 |___|

1. **Unaweza kutaja sikuzawiki kinyume kuanzia juma pili/** Can you say the days of the week backwards starting with Sunday **.** Give mark for all days given in a correct sequence (up to 5)**/Mpealama kwa kila siku iliyotajwa kwa usahihi (zisizidi alama 5** [___]

1. **Hesabu kuanzia 20 kushuka chini mpaka 1** / Count from 20 down to 1

**Siyo sawa/**Incorrect 0 **Sawa/** Correct 1 |___|

1. **Taja vitu vilivyoko kwenye picha hii (alama tano)/**Name the objects in the picture (score out of five) [___] outline images of cow (ngombe), hen (kuku), pig (nguruwe), elephant (tembo), fish (Samaki)

Family History

1. **Kuna jamaa zako yewote ambao wana tatizo la kusahau au walikuwa na tatizo la kusahau? /** Have any of your close relatives such as parents, brothers, sisters or children had the problem of serious loss of memory? **ANDIKA UMRI TUTIZO LA KUSAHAM LILIPOANZIA/**PUT AGE OF ONSET

No 0 Yes 1 [___]

Baba_____Mama_____Kaka_____Dada_____Mtoto wa kiume_____Mtoto wa kike_______

**USAHILI NA MGONJWA/INTERVIEW WITH PATIENT**

1. Maelezo ya usahili kwa ujumla. Mapendekezo (watu waliopo maendeleo ya usahili, muingihano wa muhimu mapendekezo ya kumsaidia msahili kukumbuka usahili huu) General description of interview. Comments (people present, progression of interview, significant interruptions, comments to help interviewer remember this interview).

Weka kwonyesha ni kiasi gani matatizo yafwatayo yanajitokeza/ Rate degree problem interfered with testing:

|  | None**/Hamna**  1 | Mild/**Kidogo**  2 | Moderate/**Kiasi**  3 | Severe/**Sana**  4 |
| --- | --- | --- | --- | --- |
| Deafness/**Kiziwi** |  |  |  |  |
| Problems with eyesight/**Matatizo ya macho** |  |  |  |  |
| Physical problems interfering with testing/**Matatizo ya maumbile yanayozuia uchunguzi** |  |  |  |  |

2. Describe observed physical and mental state of subject including general appearance and nutrition status. (cooperation, agitation, anxiety, etc.)/ **Elezea muonekano wa mgonjwa (anaushirikiano)**

3. Reliability of information contained in this assessment.**Taarifa hii inausahihi au ni ya kutegemea kwa kiasi gani?**

1……….Very Good/**Kwa hali ya juu**

2……………..Good/**Kwa hali ya kati**

3……..….Only Fair/**Kwa hali ya chini**

4…….………..Poor/**Haifai**

5………..Very Poor/**Haifai kabisa**

4. Completeness of this interview:**Mahojiano haya yalifikia mwisho**

1……..…**Yalimalizika/**Complete

2………**Hayakumalizika/**Incomplete

**Kama hayakufikia elezea /** If incomplete, specify: __________________

**ANAYECHUKUA TAARIFA HII ANAHISI MGONJWA ANAMATATIZO YA KUSAHAU/** DOES THE PERSON CARRYING OUT THE CSID FEEL THE PATIENT HAS DEMENTIA

**NDIYO/** YES

**HAPANA/** NO

The Community Screening Instrument for Dementia (CSI-D**)/ Njenzo Itumikayo Kutambua Tatizo la Dementia**

## B: Informant interview/Dodoso kwa (mlengwa)

Name of relative (first)__________________(middle)______________(surname)________________

**Jina la ndugu (Jinala kwanza) (Jinala katikati) (Jina la baba)**

Relationship to patient**:** Spouse/**mme/mke** 1 Sibling **mtoto** 2 Child **mtoto** 3

**Uhusiano namgonjwa** Grandchild/**mjukuu** 4 Other (specify)**menginejo (eleza)**________________5 [___]

Age (yrs) **Umri (miaka)**__________

Residence/**Makazi**: Lives with patient/**anaishi na mgonjwa** 1 Other/**menginejo** 2 Specify**/Eleza**______________ [___]

How often does the relative see the patient/**Ni mara ngapi ndugu anamuona mgonjwa:**

Every day/**Kilasiku** 1 Every other day/**Kita baada ya siku moja** 2 Once a week/**mara moja kwa wiki** 3

Once a month/**Mara moja kwa mwezi** 4 Other/**menginejo** 5 Specify/ **Eleza** ____________________ [___]

Occupation

**Ningependa kukuuliza maswali machache kuhusu shuguli za muhusika Bw/Bi________/**I would like to ask a few brief questions about Mr/Mrs _________

1. **Shuguli zake ni / zilikuwa gani? /** What was their main occupation?______________________

**1a) Kazi Maalum /** Primary occupation______________________________________________

**1b) Muda gani /** How long did they do that work, record number of years? _________________

**1c) Kazi ingine /** Secondary occupation ____________________________________________

**1d) Muda gani? /** How long did he/she do that work? Record number of years ______________

1. **Aliacha kufanya hii kazi lini? /** When did he/she stop working regularly? Yr stopped________

Still working________

1. **Kwa sababu gani alicha kazi? /** Why did he/she stop working?

**Yungali kazini /** Still working at a paying job 1

**Yungali anafanya kazi za nyumbani /** Still working at housework/ family upkeep 2

**Kustaafu /** Retired 3

**Kutojiweza –afya mbaya /** physical health problems 4

**Tatizo la akili /** mental health problems 5

**Ingine /** Other 6

**Haijulikani /** not known 7 [_­__]

Daily Activities

**Ningependa kukuuliza maswali machache kuhusu shuguli za muhusika Bw/Bi_______za siku hizi** / I would like to ask a few brief questions about your __________ activities these days.

1. **Ana shuguli gani kwa sasa? /** Currently what are his/her main activities

**Hana shuguli isipokuwa ya kibinafsi nay a nymbani akisaidiwa /** None personnel and home maintainance assisted by others 0

**Shuguli za nymbani, na kulisha wengine peke yake /** Some active at home takes care of self and other family members 1

**Shuguli za inge /** Extensive activities outside the home, attends community meetings, volunteers at church 2

**Haijulikani /** not known 3

[___]

1. **Umeona mabadiliko katika kuendesha shuguli zake za kila siku ukilinganisha na miata kadhaa iliyopita?** Have you seen a change in his/her daily activities in the past several years?

**Hakuna mabadiliko** / No change 0

**Unapungua** / slowing down 1

**Utendaji unapungua au anaacha kutokana na matatizo ya kiafya tunayoyajua** / activities decreased or discontinued due to known health problem 2

**Utendaji unapungua au anaacha kutokana na tatizo la akili**/ activities decreased or discontinued due to mental health problem 3

**Utendaji unapungua au anaacha bila sababu yeyote**/ activities decreased or discontinued no apparent reason 4

**Haijulikani**/ not known 5 [___]

1. **Je amekuwa na upungufu wa akili? Fafanua** Has there been a general decline in his/her mental functioning?

Please describe

No**/hapana** 0 Yes/**Ndio** 1 Not known/**Haijulikani** 2 |___|

**Ni lini uliona hili tatizo kwa mara ya kwanza?/Kadiria tarche**/ When did you first notice this? Estimate date ____/____/____**Ni miezi mingapi imekua ni tatizo /**Or record number of months it has been a problem_________

1. **Sisi wote tunashida kidogo ya kukumbuka vitu tunapo kuwa wazee, lakini hii imekua ni tatizo kwa huyu?** We all have slight difficulties in remembering things as we get older, but has this been a *particular* problem for x?

No**/hapana** 0 Yes/**Ndio** 1 Not known/**Haijulikani** 2 |___|

Cognitive Functioning

**Sasa ningependa kukuuliza juu ya mabadiliko yoyote ambayo umeyaona kuhusu huyu jamaa (Mke / Bwana)** Now I would like to ask you about other changes you may have noticed in your wife/husband etc

1. **Je, anasahau mahali ambapo ameweka vitu**? / Does he / she forget where she has put things?

No/**hapana** 0 Sometimes/**Si wakati wote** 0.5 Yes/**Ndio** 1 Not known/**Haijulikani** 2 |___|

1. **Je, anasahau mahali vitu huwekwa?** / Does he/she forget where things are usually kept?

No/**hapana** 0 Sometimes/**Si wakati wote** 0.5 Yes/**Ndio** 1 Not known/**Haijulikani** 2 |___|

1. **Je, yeye husahau majina ya marafiki?** / Does he/she forget the names of friends?

No/**hapana** 0 Sometimes/**Si wakati wote** 0.5 Yes/**Ndio** 1 Not known/**Haijulikani** 2 |___|

1. **Au, jamaa zake?** / Or members of the family?

No/**hapana** 0 Sometimes/**Si wakati wote** 0.5 Yes/**Ndio** 1 Not known/**Haijulikani** 2 |___|

1. **Je, anasahau maneno anayotaka kusema katikati ya majadiliano**? / Does he/she forget what they wanted to say in the middle of the conversation?

No/**hapana** 0 Sometimes/**Si wakati wote** 0.5 Yes/**Ndio** 1 Not known/**Haijulikani** 2 |___|

1. **Na akiongea, anashida ya kutamka maneno kwa usa?** / When speaking does he/she have difficulty saying the right words?

No/**hapana** 0 Sometimes/**Si wakati wote** 0.5 Yes/**Ndio** 1 Not known/**Haijulikani** 2 |___|

1. **Je, anatumia maneno yale yasiyo sahihi?** / Does he/she use the wrong words?

No/**hapana** 0 Sometimes/**Si wakati wote** 0.5 Yes/**Ndio** 1 Not known/**Haijulikani** 2 |___|

1. **Hupenda kuongea juu ya mambo ya zamani kuliko mambo ya sasa?** / Does he/she tend to talk about what happened long ago rather than the present?

No/**hapana** 0 Sometimes/**Si wakati wote** 0.5 Yes/**Ndio** 1 Not known/**Haijulikani** 2 |___|

1. **Je, yeye husahau wakati alipokuona maraya mwisho**? / Does he/she forget when she last saw you?

No/**hapana** 0 Sometimes/**Si wakati wote** 0.5 Yes/**Ndio** 1 Not known/**Haijulikani** 2 |___|

1. **Je, yeye husahau yaliyotendeka jana?** / Does he/she forget what happened the day before?

No/**hapana** 0 Sometimes/**Si wakati wote** 0.5 Yes/**Ndio** 1 Not known/**Haijulikani** 2 |___|

1. **Huwa anasahau mahali yupo?** / Does he/she forget where she is?

No/**hapana** 0 Sometimes/**Si wakati wote** 0.5 Yes/**Ndio** 1 Not known/**Haijulikani** 2 |___|

1. **Huwa anapotea kijijini (mfano kwenda dukani au sokoni)?** / Does he/she get lost in the village? E.g. finding the shop or the market?

No/**hapana** 0 Sometimes/**Si wakati wote** 0.5 Yes/**Ndio** 1 Not known/**Haijulikani** 2 |___|

1. **Huwa anapotea nyumbani kwake (k.m. mahali choo ilipo)?** / Does he/she get lost in his / her own home, e.g finding the toilet?

No/**hapana** 0 Sometimes/**Si wakati wote** 0.5 Yes/**Ndio** 1 Not known/**Haijulikani** 2 |___|

Activities of Daily Living

1. **Anashida kufanya zile kazi za nyumbani alizokuwa akifanya hapo nyuma? (kupika, kufuga wanyama)**Does he/she have difficulty performing household chores that he/she used to do, e.g. preparing food or tying the animals?

No/**hapana** 0 Sometimes/**si wakati wote** 0.5 Yes/**ndiyo** 1 Not known/ **Haijulikani** 2 |___|

21a. Does the interviewer think that the problem is primarily due to physical disability?

**Hapana**/No 0 **Ndio**/Yes 1 **Haijulikani**/Not known 2 |___|

1. **Amepoteza ujuzi wuwote aliokuwa nao mbeleni (mfano kushona, kucheza)?** / Has there been a loss of a special skill or hobby he/she could manage before?

**Hapana**/No 0 **Ndio**/Yes 1 **Haijulikani**/Not known 2 |___|

22a. Does the interviewer think that the problem is primarily due to physical disability?

**Hapana**/No 0 **Ndio**/Yes 1 **Haijulikani**/Not known 2 |___|

1. **Ameonyesha mabadiliko katika njia ya kutumia pesa?** / Has there been a change in his/her ability to handle money?

**Hakuna ugomu/**No difficulty 0 **kuna ugumu kidogo/**Some difficulty 1 **hawezi kutukia hela/**Cannot handle money 1 **haijulikani/**Not known 2 |___|

1. **Anapata shida kutika kubadili shuguli zake za kila siku?** / Does he/she have difficulty in adjusting to change in her daily routine?

**Hapana/**No 0 **Si wakati wote/**Sometimes 0.5 **Ndio/**Yes 1 **Haijulikani/**Not known 2 |___|

1. **Je, umeona kama amebadilika uwezo wake wa kufikiri na kuamua kwa usahili**? / Have you noticed a change in his/her ability to think and reason?

**Hapana/**No 0 **Si wakati wote/**Sometimes 0.5 **Ndio/**Yes 1 **Haijulikani/**Not known 2 |___|

1. **Amekuwa na shida kujilisha (kula)?** / Does he/she have difficulty eating?

**Anakula vizuri akitumia (mikono)** / Eats cleanly with 0

**Hatumii mikono vizuri** / Eats messily 1

**Ana shida akila vitu kama karanga** / Simple solids such as groundnuts 2

**Lazima alishwe** / Has to be fed 3

**Haijulikani** / Not known 4 |___|

**26a**. **Mdodosaji anafikiri kuwa tatizo himesababishwa na ulemavu unavonekana?**Does the interviewer think that the problem is primarily due to physical disability?

**Hapana**/No 0 **Ndio**/Yes 1 **Haijulikani**/Not known 2 |___|

1. **Anashida ya kuvaa nguo?** / Does he/she have difficulty dressing?

**Anavaa yeye mwenyewe** / Dresses self 0

**Wakati mwingine anafunga vifungo vibaya** / Occasionally misplaces buttons etc. 1

**Hana mtiriko mauri, mara nyingi husahau vitu** / Wrong sequences, commonly forgets items 2

**Hawezi kuvaa** / Unable to dress 3

**Haijulikani** / Not known

4 |___|

27a. **Mdodosaji anafikiri kuwa tatizo himesababishwa na ulemavu unavonekana?**Does the interviewer think that the problem is primarily due to physical disability

**Hapana**/No 0 **Ndio**/Yes 1 **Haijulikani**/Not known 2 |___|

1. **Ana shida ya kutumia choo?** **/ Anajikojolea au kujiloanisla mwenyewe?** Does he/she have difficulty using the toilet? / Does he/she wet or soil herself?

**Hana shida** / No problems 0

**Wakati mwingine hukojoa kitandani** / Occasionally wets bed 1

**Hukojoa kitandani kila mara** / Frequently wets bed 2

**Hujikojolea na hujinyea** / Double incontinence 3

**Haijulikani** / Not known 4 |___|

28a. **Mdodosaji anafikiri kuwa tatizo himesababishwa na ulemavu unavonekana?**Does the interviewer think that the problem is primarily due to physical disability

**Hapana**/No 0 **Ndio**/Yes 1 **Haijulikani**/Not known 2 |___|

Personality

**Sasa ningependa kujua kama Bw/Bi ____________ amekuwa na mabadiliko yoyote kwa vitendo vyake.** Now I would like to know about any changes in Mr/Mrs______________personality

1. **Umeona mabadiliko yoyote kwa vitendo vyake? /** Have you noticed any changes in his / her personality

**Hapana**/No 0 **Ndio**/Yes 1 **Haijulikani**/Not known 2 |___|

**Fafanua /** Describe_____________________________________________________________________________

1. **Amekuwa anakasirika kwa urahisi? /** Has he/she become more irritable?

**Hapana**/No 0 **Ndio**/Yes 1 **Haijulikani**/Not known 2 |___|

1. **Amekuwa akisumbua? /** Has he/she become more stubborn?

**Hapana**/No 0 **Ndio**/Yes 1 **Haijulikani**/Not known 2 |___|

1. **Amewacha kujali masilahi ya watu wengine? / Does he/she show less concern for other people?**

**Hapana**/No 0 **Ndio**/Yes 1 **Haijulikani**/Not known 2 |___|

1. **Je, amepoteza furaha katika maisha?** / Is there a loss of interest or enjoyment in things in general?

**Hapana**/No 0 **Ndio**/Yes 1 **Haijulikani**/Not known 2 |___|

1. **Amepoteza furaha kwa vitu alivyokuwa akipenda kitambo?** / Has he/she lost interest in things he/she used to enjoy?

**Hapana**/No 0 **Ndio**/Yes 1 **Haijulikani**/Not known 2 |___|

1. **Je, unaona ana huzuni nyingi zaidi kuliko hapo zamani? (fafanua) /** Do you think he/she is more depressed than used to be? (describe evidence)

**Hapana**/No 0 **Ndio**/Yes 1 **Haijulikani**/Not known 2 |___|

___________________________________________________________________________________

1. **Je, unaona ana wasi wasi zaidi siku hizi? (fafanua dalili) /** Do you think he/she is more nervous than he/she used to be? (describe evidence)

**Hapana**/No 0 **Ndio**/Yes 1 **Haijulikani**/Not known 2 |___|

___________________________________________________________________________________

1. **Unajua kama kuna jamaa yake yeyote amewahi kuwa na tatizo la kusahau? /** Do you know if any of his / her close relatives such as parent, brothers, sisters, or children have had the problem of extreme loss of memory?

**Hapana/**No 0 **Ndio/**Yes 1 [___]

Father______ Mother_____ brother_____ sister______ son_______ daughter______

**Interviewer’s Notes - Interview with the relative**

**Maelezo ya msahili – usahili na ndugu. Maelezo ya usahili kwa ujumla. Mapendekezo (watu waliopo, Maendeleo ya usahili, muingiliano wa muhimu, mapendekezo ya kumsaidia msahihi kukumbuka usahili huu)** General Description of the interview. Comments (people present, progression of interview, significant interruptions, comments to help interviewer remember this interview)

**Taarifa hii ni ya kutegemea kwa kiasi gani?** Reliability of the information contained in this assessment.

**Kwa hali ya juu/**Very good 1

**Kwa hali ya kati/**good 2

**Kwa hali ya chini** /fair 3

**Haifai**/poor 4

**Haifai Kabisa**/ very poor 5 [__]

**Usahili huu ulifikia /** Completeness of this interview:

**Ulimalizika/** Complete 1

**Haukumalizika/**incomplete 2 [__]

**Iwapo haukumalizika elezea ni kwa nini?** If incomplete, why: __________________________________________________________________________________________

**CERAD 10 word learning list/ Orodha ya maneno kumi ya kujifunza**

**Tafadhali msome mgonjwa maneno yote kwenye orodha alafu mwambie mgonjwa ajaribu kwa mara ya kwanza kumdia atakayoweza kukumbuka. Tafadhali fanya zuezi la mwisho uliza tena (kwa kuringalia kumbukumbu ya baadae)**

| **Swahili** | **Jaribio la kwanza** | **Jaribio la pili** | **Jaribio la tatu** | **Kumbukumbu ya baadae** |
| --- | --- | --- | --- | --- |
| Siagi |  |  |  |  |
| Mkono |  |  |  |  |
| Barua |  |  |  |  |
| Mfalme |  |  |  |  |
| Tikiti |  |  |  |  |
| Nyasi |  |  |  |  |
| Kona |  |  |  |  |
| Jiwe |  |  |  |  |
| Kitabu |  |  |  |  |
| Fimbo |  |  |  |  |

**Tafadhali andika matatizo yoyote ya kiafya:**
